# Molecular, phenotypic, and sample-associated data to describe pluripotent stem cell lines and derivatives

**DOI:** 10.1038/sdata.2017.30

**Published:** 2017-03-28

**Authors:** Kenneth Daily, Shannan J. Ho Sui, Lynn M. Schriml, Phillip J. Dexheimer, Nathan Salomonis, Robin Schroll, Stacy Bush, Mehdi Keddache, Christopher Mayhew, Samad Lotia, Thanneer M. Perumal, Kristen Dang, Lorena Pantano, Alexander R. Pico, Elke Grassman, Diana Nordling, Winston Hide, Antonis K. Hatzopoulos, Punam Malik, Jose A. Cancelas, Carolyn Lutzko, Bruce J. Aronow, Larsson Omberg

**Affiliations:** 1 Sage Bionetworks, Seattle, Washington 98109, USA; 2 Department of Biostatistics, Harvard T.H. Chan School of Public Health, Boston, Massachusetts 02115, USA; 3 Institute for Genome Sciences and Department of Epidemiology and Public Health, University of Maryland School of Medicine, Baltimore, Maryland 21201, USA; 4 Department of Biomedical Informatics, Cincinnati Children’s Hospital, Cincinnati, Ohio 45229, USA; 5 Division of Experimental Hematology and Cancer Biology, Cincinnati Children’s Hospital, Cincinnati, Ohio 45229, USA; 6 Division of Human Genetics, Cincinnati Children’s Hospital, Cincinnati, Ohio 45229, USA; 7 Division of Developmental Biology, Cincinnati Children’s Hospital, Cincinnati, Ohio 45229, USA; 8 Gladstone Institutes, San Francisco, California 94158, USA; 9 Translational Trials Development and Support Laboratory Field Service, Cincinnati Children’s Hospital, Cincinnati, Ohio 45229, USA; 10 Cell Manipulation Laboratory, Cincinnati Children’s Hospital, Cincinnati, Ohio 45229, USA; 11 Sheffield Institute for Translational Neuroscience, University of Sheffield, Sheffield S10 2HQ, UK; 12 Harvard Stem Cell Institute, Cambridge, Massachusetts 02138, USA; 13 Departments of Medicine and Cell and Developmental Biology, Division of Cardiovascular Medicine, Vanderbilt University, Nashville, Tennessee 37232, USA

**Keywords:** Data integration, RNA sequencing, Methylation analysis, Differentiation, Induced pluripotent stem cells

## Abstract

The use of induced pluripotent stem cells (iPSC) derived from independent patients and sources holds considerable promise to improve the understanding of development and disease. However, optimized use of iPSC depends on our ability to develop methods to efficiently qualify cell lines and protocols, monitor genetic stability, and evaluate self-renewal and differentiation potential. To accomplish these goals, 57 stem cell lines from 10 laboratories were differentiated to 7 different states, resulting in 248 analyzed samples. Cell lines were differentiated and characterized at a central laboratory using standardized cell culture methodologies, protocols, and metadata descriptors. Stem cell and derived differentiated lines were characterized using RNA-seq, miRNA-seq, copy number arrays, DNA methylation arrays, flow cytometry, and molecular histology. All materials, including raw data, metadata, analysis and processing code, and methodological and provenance documentation are publicly available for re-use and interactive exploration at https://www.synapse.org/pcbc. The goal is to provide data that can improve our ability to robustly and reproducibly use human pluripotent stem cells to understand development and disease.

## Background & Summary

The development of methods to transform adult somatic cells into induced pluripotent stem cells (iPSC) have lead to new opportunities for disease modeling and clinical translation^1–3^. While methods of somatic cell reprogramming are being continually improved^4,5^, prior to transplantation of iPSC or their differentiated derivatives, extensive characterization of each line must be performed to ensure normal growth characteristics, pluripotentiality, genetic stability and lack of immunoreactivity. For comparison purposes, human embryonic stem cells (hESC) have been considered to be the functional, genetic and epigenetic ‘gold standard’ in this field^6^. Such analyses are also needed to ensure the reproducible use of existing iPSC lines for laboratory research purposes.

**Figure 1:**
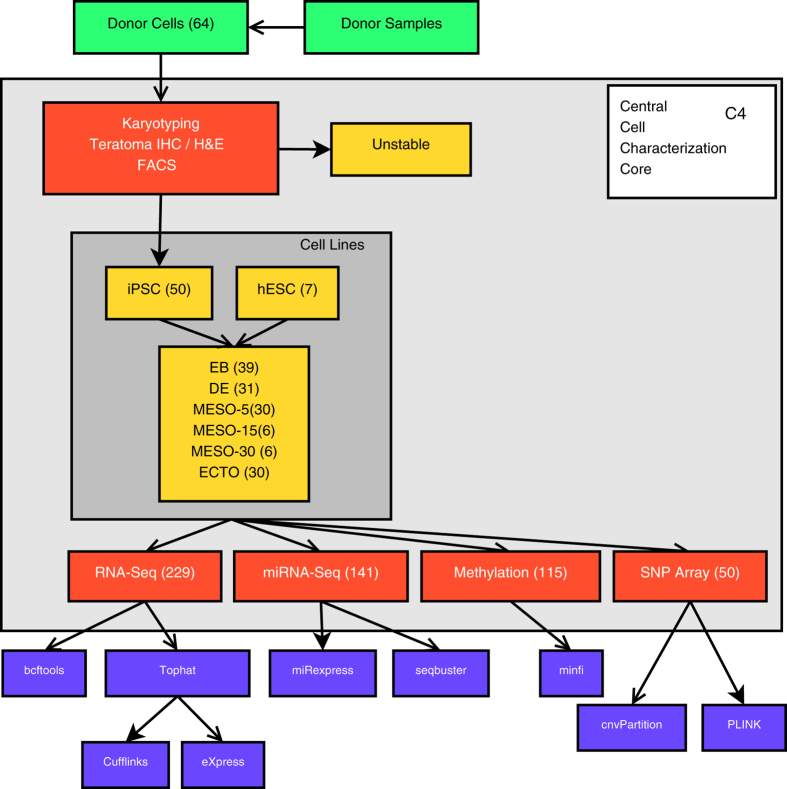
Integrated workflow for stem cell characterization and data integration. The types of cell lines evaluated by the PCBC Cell Characterization Core, assays and data processing pipelines are indicated. Numbers in parentheses indicate number of distinct cell line samples, except at assay level, where they indicate the number of individual assays (including replicates).

The Progenitor Cell Biology Consortium (PCBC) of the National Heart, Lung and Blood Institute was established to investigate and optimize methods for reprogramming and differentiation of iPSC as a precursor to transplantation studies with these lines. The 64 stem cell lines (58 iPSC and 6 hESC) selected were obtained from a diverse set of somatic cell of origins, gene reprogramming combinations, culturing methods (e.g., stromal priming) and reprogramming vectors for both distinct and common genetic donors. These lines were sent to a centralized core laboratory for cell culturing, mycoplasma testing, growth characterization, flow cytometry analysis, and *in vivo* and *in vitro* differentiation potential. To provide an unbiased evaluation of PSC stability and quality, we recently characterized 57 of these PSC lines using a broad range of phenotypic and molecular omics assays^7^. A single characterization core laboratory was used to ensure sufficient standardization of these methods. After these characterizations, 57 cell lines were differentiated into 7 different states, including 3 germ layers and embryoid bodies, and subsequently characterized using genomic assays (CNV, DNA-methylation, mRNA and microRNA) (Fig. 1).

To ensure the comparability of all identifiable covariates, we developed descriptive metadata standards, including ontology defined controlled vocabularies in addition to consistent quality control metrics and data analysis methods. All relevant documentation and data has been deposited in Synapse (https://www.synapse.org/pcbc) (Data Citation 1), a publicly available online collaborative research platform that provides data annotation, documentation, and file provenance^8^. Specifically, we deposited metadata, *in vitro* and *in vivo* differentiation, qPCR, RNA-seq, miRNA-seq, copy number variation, and DNA methylation data, and processed results from both low- and high-throughput analyses. An interactive browser was developed for querying, filtering, analyzing, and visualizing the genomics data. For users looking to reprocess the raw data, we provide annotations for querying and automatically downloading all raw and intermediate data files. We are also using the portal to distribute insights and results of the analysis as they become available. The data provided is available for unrestricted reuse. We encourage other researchers and members of the public to download and critically analyze this resource.

## Methods

The PCBC Central Cell Characterization Core (C4) established standard protocols for sample collection, handling, and analysis (syn2512369) and the PCBC Bioinformatics Core established standard data processing techniques for the multi-omic data as well as computational quality control. The details of experimental methods for the handling and processing of the stem cells used here have been previously described^7^.

### Standardization of cell line metadata

The PCBC Bioinformatics Committee Working Group coordinated the development and implementation of a PCBC cell line characterization metadata standard (syn2767699) for the initially donated cell lines. Through an iterative process, cell line characterization metadata terms were defined and mapped to Open Biological and Biomedical Ontologies (OBO) Foundry ontologies^9^ available through the National Center for Biomedical Ontology’s (NCBO) BioPortal^10^. When ontology terms were not sufficiently defined in their source ontologies, new terms were defined and requested from ontology editors. For example, cell type terms from the Cell Ontology such as ‘endothelial stem cell’ were added to describe the cell line type, and cell terms from UBERON were added to describe the cell line’s tissue of origin.

The collected metadata includes detailed information on each associated cell line collected from the submitting investigators including cell of origin, method of reprogramming, reprogramming gene combination, donor sex, ethnicity and disease status (syn2767694). Metadata information was initially provided by the originating laboratory, and was subsequently augmented with *in vitro* genetic and experimental characterization data of the line (such as karyotype status), and resubmitted to the originating lab for confirmation. Metadata fields have also been added to facilitate sample comparisons in downstream analyses (see the Usage Notes).

### Sample collection and handling

Multiple institutions contributed cell lines used in this study, all of which were generated using IRB-approved protocols from the initiating institution. Approval letters or designation of non-human subjects research (for some made with waste products) were received from all institutional IRB. Since no identifying information on the lines was provided to the C4, this study was performed under an Embryonic Stem Cell Research Oversight Committee (ESCRO) approval, as it was not considered human subjects research, and under the IRB at Cincinnati Children's Hospital Research Center.

In brief, hESC and iPSC lines were cultured (using protocols syn2724700 and syn2724705) and stored (using protocol syn2724707). Each stem cell line was also evaluated in a teratoma assay (protocol syn3103753) and karyotyped. Reports and images for the teratoma assays (syn2882774) and karyotyping (syn2679104) are available. The lines were then differentiated into germ layer states: ectoderm, definitive endoderm, and mesoderm. The mesoderm differentiation protocol (syn2512371) was adapted from Zhang *et al.*^11^, and samples were taken at 5, 15, and 30 days after differentiation. The ectoderm differentiation protocol (syn2512373) was adapted from Chambers *et al.*^12^. The definitive endoderm differentiation protocol (syn2512372) was adapted from D’Amour *et al.*^13^. Cells were additionally differentiated in embryoid body (EB) cultures for 17 days. On day 7, they were grown out from the EB using the embryoid body differentiation protocol (syn2512370). After differentiation, cells were harvested for RNA and DNA extraction.

### Genomic and epigenetic molecular characterization

In brief, gene and microRNA expression were assessed by Illumina HiSeq 2,000 sequencing, and methylation was assessed with the Illumina HumanMethylation450 BeadChip. Two assays were used for copy number variation analysis: 21 cell lines were assayed with the Illumina CytoSNP-850K BeadChip, and 29 cell lines were assayed with the Illumina HD HumanOMNI-Quad BeadChip platform. For validation of gene expression, thirty-seven samples were analyzed using a TaqMan Low Density Array (TLDA) (Stem Cell Pluripotency Array, 43,85,344, Life Technologies) that interogates a panel of stem cell and pluripotency marker genes (syn3107327).

### Data processing

Each assay was processed through one or more reproducible pipelines. Details of the processing for each file is stored in Synapse as part of a provenance record for the file, which links back to code, input files, and parameters. Prior to processing, samples with quality control issues (described in the subsequent assay processing descriptions) were excluded.

### RNA-Seq data processing

All samples were evaluated for a variety of quality control metrics including alignment percentage, proportion of exonic reads, and distribution of reads at the 5′ and 3′ ends of transcripts using the Cincinnati Children’s Medical Center DNA sequencing core automated pipeline. Samples with quality control issues (e.g., poor 5′ to 3′ ratios or abnormal karyotype) were flagged in the metadata and were not included in downstream analyses. FASTQ files (syn1773112) were aligned to the human genome build GRCh37 and University of California Santa Cruz (UCSC) transcriptome ref. 14 (syn5663983) using Tophat 2.0.9 (ref. 15). Quantification was performed using two methods. Gene-level reads per kilobase per million reads (RPKM) values were quantified using Cufflinks 2.0.2 (refs 16,17) with the Ensembl transcriptome reference (syn5664028) using corrections for sequence-specific bias and multi-mapped reads (syn2247799). Also, eXpress 1.4.0 (ref. 18) was used to quantify the transcript level (syn3270268) using UCSC’s knownGene transcriptome reference (syn3351175). The resulting transcript quantifications were summed over transcripts to the gene level for downstream analyses (syn5008587). Alternative splicing estimates were obtained using AltAnalyze^18,19^. For AltAnalyze analysis, unique putative novel exons were determined from all Tophat junction alignments in AltAnalyze version 2.0.9 (ref. 19) (http://www.altanalyze.org) and analyzed for associated exon-read coverage using the BedTools function BAMtoBED^20^, along with all AltAnalyze predicted exons (Ensembl 72 and UCSC annotated mRNAs). The resulting exon and junction BED files for AltAnalyze were used as input for downstream statistical and visualization analyses (clustering, PCA, and network analysis) in AltAnalyze, using indicated stringency options for transcription, exon and reciprocal junction analyses (syn3105745). These analyses include reciprocal junction Percent Spliced In (PSI) analysis, in which the ratio of junction read counts was calculated for each evaluated exon-exon junction compared to the total number of junction read counts for all genomic overlapping TopHat detected exon-exon junctions (known and novel). Samples without sufficient junction read-depth (>4 reads) for each evaluated splicing event were not considered for statistical analyses. For splicing visualization, coverage plots were produced from the Broad’s IGV Sashimi-Plot function^20,21^.

### miRNA-Seq data processing

miRNA samples were assessed for quality and samples marked as `exclude` had undiagnosed difficulties during sequencing (e.g., only a few miRNAs with aligning reads) and were not used in downstream analyses. miRNA expression was quantified with mirExpress v2.1.4^22^ using the human miRBase 20.0 (ref. 23) (syn2247097). miRNA expression was also quantified with seqbuster^24^ (syn3355992) using the human miRBase 21.0 (ref. 23) (syn6185321) of 2,588 annotated miRNAs. The counts for each microRNA was further filtered to 2,303 miRNAs with at least 2 reads aligned in each mature miRNA. Samples were considered failing QC that had low overall sequencing read depth of lower than 0.5 million reads and the percentage of annotated miRNA lower than 20%.

### DNA methylation data processing

DNA-methylation arrays were normalized with the minfi R package^25^ (syn2233188). 15 samples were removed due to poor intensity identified by minfiQC. The remaining samples were quantile normalized and inspected using principal components analysis.

### CNV analysis

The plug-in cnvPartition (v3.2.0) for GenomeStudio was used to identify CNVs from the SNP arrays. For this software, the default settings were used, with the exception of a minimum loss of heterozygosity was changed to 5 Mb and minimum number of SNPs changed to 10. Microarray copy number final reports were generated (syn2679103).

### Genetic analysis

SNPs from the SNP arrays were augmented with SNPs called from RNA-Seq for quality control purposes. SNPs were called from from RNA-Seq using SamTools 0.1.18 (ref. 26) and subsequently filtered for read depth using vcftools^27^ (syn2390898).

## Data Records

### Data availability overview

All associated data described in this manuscript are available for download at http://www.synapse.org/pcbc (Data Citation 1). All computationally derived files also include provenance, a historical record of how, when, and by whom they were generated, which provides the ability to understand the origin of the results, reproduce them, apply the same procedures to new data, and attribute the work performed. The main Project page includes details about the PCBC, overviews of the available data and analyses, and links to main sections of content. Many individual files and folders have their own Wiki content as the equivalent of a ‘README’ file, providing context and detailed information directly with the content. In addition to data files, the analysis code and workflows for all associated files has been included where appropriate. Analysis scripts are also provided through provenance records tracked with individual files. The code and scripts are hosted in Github (https://www.github.com/Sage-Bionetworks/pcbc_c4_analysis) and are available for reuse under the MIT license. Synapse accession numbers (in the form synXXXX) are listed with specific files and folders directly in the manuscript. Each record also has an associated Document Object Identifier (DOI) (in the form http://doi.org/10.7303/synXXXXX) that can be used for citation. Each assay (mRNA, miRNA, and methylation) has an associated Folder that contains all raw data files (Table 1). Each folder has a Wiki describing the contents and providing direct links to data files processed through each assay’s data processing pipeline. Raw data is also available from the NCBI through BioProject Accession PRJNA338817 (Data Citation 2). High throughput sequencing data for mRNA and miRNA is available from the Short Read Archive (Data Citation 3), and DNA methylation microarray data is available from GEO (Data Citation 4). Data and metadata generated by the C4 are available under a Creative Commons CC0 1.0 Universal license (https://creativecommons.org/publicdomain/zero/1.0/), meaning they can be used for any purpose without restriction. See the C4 data page (https://www.synapse.org/#!Synapse:syn1773109/wiki/218833) for further information.

To aid in interactive analyses of this data, a data exploration tool has been developed to facilitate gene-level and cluster visualization as well as functional enrichment analyses using ToppGene^28^ This tool is freely available (with a Synapse account) at https://www.synapse.org/#!Synapse:syn1773109/wiki/63531.

### Metadata collection and standardization

The PCBC cell line metadata standard containing over 100 terms is available (syn2767699). Term definitions and URLs for term sources are included. In brief, the top level of terms in each controlled vocabulary (CV) include: PCBC cell line name, originating laboratory ID, C4 cell line ID, host species, cell line type, somatic cell type, iPSC cell type of origin, tissue of origin, reprogramming vector type, induced reprogramming gene combination, culture conditions, donor age, donor life stage, race, ethnicity, sex, disease, donor phenotype, reprogramming laboratory, and PCBC primary investigator.

Each PCBC cell line term has been defined and linked to an OBO Foundry ontology term, where available. Ontologies include the NCBI Taxonomy CV, Cell Line Ontology, Cell Ontology, SO: Sequence Types and Features Ontology, NCI Thesaurus, HsapDv, PATO, the Disease Ontology and the Human Phenotype Ontology. The metadata characterization of the lines that are undergoing or completed characterization are available in Synapse (syn2767694). Complete metadata, including specifics about the assays (DNA and RNA isloation dates, alignment statistics, etc.) for each assay are also available: mRNA (syn3156503), miRNA (syn3219876), and methylation (syn3156828).

All assay-related files have a minimum set of annotations to facilitate programmatic access. First, all assay files have a unique identifier (UID) for the specific assay (generally a combination of the cell line, differentiation state, replicate, and assay run information). This UID matches up to the records in the assay metadata tables described above and are used to automate the annotation of the individual files. Other annotations are manually added at the time of upload into Synapse. The `dataType` annotation notes which assay the data file comes from: mRNA, miRNA, methylation. The `fileType` annotation denotes the type of file (e.g., align, bam, bed, count, expr, fastq, fpkm, genomicMatrix, idat, report). Many files also have a file sub-type (e.g., exons, deletions, gene, insertions, isoform, junctions, mapped, unmapped, channel). These annotations can be used to query for specific sets of files, as described below in the Usage Notes section. This provides the abiliity to easily query (using the Synapse clients) for files for a specific cell line, reprogramming vector, or any other attribute described in the metadata tables. Files for a specific cell line across assays can also be identified (using the dataType annotation) or for a specific part of the pipeline process (using the fileType annotation, e.g. ‘bam’ for aligned reads in the BAM format). Example queries are provided below in the Usage Notes section.

### Experimental protocols

Experimental protocols used in the processing of C4 cell lines are available for public use (syn2512369). These include protocols for handling, storing, and differentiating stem cells. Furthermore, 5 hub sites of the PCBC have also contributed 23 protocols used in their own laboratories for the processing, handling, and induction of stem cells. Each of these protocols are annotated with the lab from where the samples originated and, if available, the individual scientists who the protocol should be attributed to. Each protocol has a DOI so future use of the protocol can be cited and attributed to the lab and individuals responsible for it’s creation.

### Cell line pages

We have automatically generated curated pages (syn5762789) for each of the publicly available C4 cell lines which contains cell line and assay metadata as well as summaries and tables of links to all raw and derived files for the specific line in Synapse. Each page has a DOI for use in citation. Physical samples of cell lines have been depositied with WiCell Research Institute (www.wicell.org). Links to WiCell are being generated to be able to order the cell lines, as well as links from WiCell back to Synapse for access to derived data.

## Technical Validation

### Confirmation of cell line donor

Donor gender was confirmed by karyotyping (syn2679104), and lines originating from a common donor were identified using SNPs from arrays and RNA-Seq (see Methods) through an identity-by-descent analysis with PLINK^29^, which identified three samples with unannotated common donors (syn6185100).

### Validation of genomics characterization

**Figure 2:**
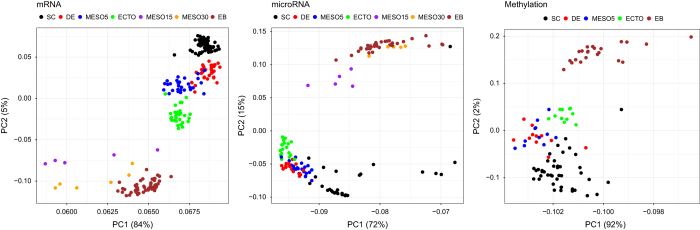
Principal components analysis of mRNA, miRNA, and methylation features. The first two PCs are shown, with sample points colored by the differentiation state.

All samples were evaluated for a variety of quality control metrics including alignment percentage, proportion of exonic reads, and distribution of reads at the 5′ and 3′ ends of transcripts using the Cincinnati Children’s Medical Center DNA sequencing core automated pipeline. Outlier samples with poor 5′ to 3′ ratios or other clear quality control issues were flagged and were not included in downstream analyses. For the remaining samples we performed an assesment of the technical and biological signals to both assess samples affected by significant technical issues (e.g., sequence read depth, RNA-quality, probe detection *P*-values) as well as determine covariates that would contribute significantly to any downstream analysis (mRNA: syn5008937; miRNA: syn5014447; methylation: syn4486559). We utilized principal component analysis (PCA) and hierarchical clustering as a primary modes of assessing potential variability and major class differences among each of the analyses differentiation states (Fig. 2).

In general, these analyses indicate that there is relative consistency in the expression and methylation profiles obtained from these diverse datasets, as best indicated by PCA. The largest degree of variability is associated with the differentiation state of the cells with contributions from patient gender, type of cell line (hESC versus iPSC) and specific donor among other signals. These analyses indicate that samples are most similar among differentiation states, suggesting this data is suitable for covariate associated comparison analyses.

## Usage Notes

### Data access and navigation

There are multiple routes for accessing the stored data: 1) web-based file navigation, 2) programmatic access (Python, R, command line, or Java), 3) interactive gene queries and visualization, and 4) signature enrichment analysis in ToppGene. All data files can be accessed through the Synapse web interface to select individual files or directories of files containing the described materials. All files contain provenance tracking information associating each result file with its associated raw formatted file, analysis scripts used for processing, version of software used, protocols, and metadata. This same data can be accessed using the Synapse clients (https://github.com/Sage-Bionetworks/synapsePythonClient, https://github.com/Sage-Bionetworks/rSynapseClient/) via the indicated Synapse accession numbers or by querying the associated metadata annotations. Examples are included for quality control and normalization of this data using IPython Notebooks and R Markdown documents for many of the provided accession numbers. Documentation about Synapse, including the clients, is available at http://docs.synapse.org/.

### Interactive and exploratory analyses

**Figure 3:**
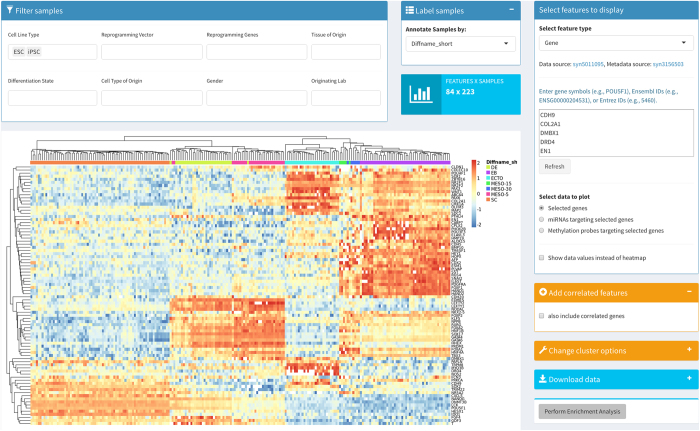
Screenshot of the interactive data explorer of the PCBC data. The mRNA, miRNA, and methylation data, along with the associated metadata, are queryable. Users can also search across assays (for example, miRNAs that may target genes). Data can be downloaded for further use.

An online application using the R package ‘shiny’ has been developed for interactive exploration of gene, miRNA or DNA-methylation signatures (https://www.synapse.org/#!Synapse:syn1773109/wiki/63531) (Fig. 3). We provide the ability to filter data based on metadata terms (cell type, reprogramming conditions, differentiation state, etc.) as well as on features (genes, miRNAs, or methylation probes). The main visualization of the data is a heatmap of expression values (or probe intensities for methylation arrays). We use publicly available records of interactions between genes and miRNAs (syn3461627), genes and methylation probes (syn2775255), and miRNAs and methylation probes (syn4895962) to link together different assays. For example, a user can provide a list of genes and visualize the expression patterns of the miRNAs that potentially target them; or, by providing a list of miRNAs, vizualize the expression of the potential target genes. Features with correlated expression patterns with the user-selected features can be optionally included automatically through a user-defined threshold of similarity. We include the ability to select gene sets based on pathways from KEGG^30,31^ and Reactome^32,33^. The heatmap can be customized using user-adjustable options for the clustering of samples or features. The underlying expression values can be directly viewed or downloaded for further analysis as text-based comma separated value files. Lastly, a direct link to the software suite ToppGene^28^ for gene set enrichment analysis of user-selected features is provided. The R code for the Shiny application is also freely available for reuse and modification at https://github.com/Sage-Bionetworks/PCBCDataExplorer.

### Querying Synapse

All assay files in this project are annotated to facilitate finding them with the information from the metadata tables. For example to find all mRNA fastq files originating from CD34+ cells (restricted to the PCBC project, syn1773109), we use a Synapse query from one of the clients. For example, from the command line client (which comes with the Python client):

synapse  query "select * from syn7511263 where dataType=mRNA AND fileType=fastq AND Cell_Type_of_Origin='CD34+ cells'"

Similarly in Python:

import synapseclient

syn = synapseclient.login()

results = syn.tableQuery("select * from syn7511263 where dataType=mRNA AND fileType=fastq AND

Cell_Type_of_Origin='CD34+ cells'")

or in the R client:

library(synapseClient)

synapseLogin()

results <- synTableQuery("select * from syn7511263 where

dataType=mRNA AND fileType=fastq AND

Cell_Type_of_Origin='CD34+ cells'")

### Data availability

The raw and normalized data products are provided as open-access data without any usage constraints or licensing required, as per the Synapse end-user access agreement. A free Synapse account (https://www.synapse.org/register) is required for downloading the available files.

## Additional Information

**How to cite this article:** Daily, K. *et al.* Molecular, phenotypic, and sample-associated data to describe pluripotent stem cell lines and derivatives. *Sci. Data* 4:170030 doi: 10.1038/sdata.2017.30 (2017).

**Publisher’s note:** Springer Nature remains neutral with regard to jurisdictional claims in published maps and institutional affiliations.

## Supplementary Material



## Figures and Tables

**Table 1:** High level organization of available raw and processed data folders on Synapse for PCBC samples.

**Assay**	**Accession**	**Description**
mRNA	syn1773112	Raw fastq
	syn1773111	Tophat BAM
	syn2246521	Cufflinks FPKM
	syn3270268	eXpress
	syn2822494	htseq-count
	syn2247799	Summarized FPKM matrix
	syn2247543	Summarized eXpress matrices
miRNA	syn2247098	Raw fastq
	syn2247164	mirExpress
	syn2247832	Summarized mirExpress counts matrix
	syn5014443	Summarized seqBuster counts matrix
methylation	syn2653626	Raw idat
	syn2233188	Summarized beta value matrix
